# Amelioration of Scopolamine-Induced Amnesic, Anxiolytic and Antidepressant Effects of *Ficus benghalensis* in Behavioral Experimental Models

**DOI:** 10.3390/medicina56030144

**Published:** 2020-03-23

**Authors:** Humna Malik, Sana Javaid, Muhammad Fawad Rasool, Noreen Samad, Syed Rizwan Ahamad, Faleh Alqahtani, Imran Imran

**Affiliations:** 1 Department of Pharmacology, Faculty of Pharmacy, Bahauddin Zakariya University, Multan 60800, Pakistan; humna.ang@hotmail.com (H.M.); sana.javaid@wum.edu.pk (S.J.); 2 Department of Pharmacy, The Women University, Multan 60000, Pakistan; 3 Department of Pharmacy Practice, Faculty of Pharmacy, Bahauddin Zakariya University, Multan 60800, Pakistan; fawadrasool@bzu.edu.pk; 4 Department of Biochemistry, Faculty of Science, Bahauddin Zakariya University, Multan 60800, Pakistan; noreen.samad@bzu.edu.pk; 5 Central laboratory, Department of Pharmaceutical Chemistry, College of Pharmacy, King Saud University, Riyadh 11451, Saudi Arabia; srahamad@ksu.edu.sa; 6 Department of Pharmacology and Toxicology, College of Pharmacy, King Saud University, Riyadh 11451, Saudi Arabia

**Keywords:** amnesia, anxiety, depression, Morris water maze, escape latency

## Abstract

*Background and Objectives: Ficus benghalensis* (FB) is a commonly found tree in Pakistan and its various parts have folkloric importance in managing neurological ailments. In the present study, methanolic extract of its bark has been tested on an experimental animal model to evaluate memory-enhancing, anxiolytic and antidepressant activities to validate the claimed therapeutic potential. *Materials and Methods:* Methanolic extract of freshly isolated bark was prepared and subjected to preliminary phytochemical studies and gas chromatography–mass spectrometry (GC–MS) analysis for the presence of phytocomponents. To evaluate its effect on spatial learning, passive-avoidance test–step through (PAT-ST), Y-maze and Morris water maze (MWM) tests were carried out. Open-field (OFT) and elevated plus maze (EPM) tests were employed to explore the anti-anxiety potential of FB while a forced swimming test (FST) was utilized to assess its anti-depressant prospective. FB doses of 100, 200 and 300 mg/kg with positive and negative controls given to Sprague Dawley (SD) rats. *Results:* phytochemical studies showed the presence of various phytoconstituents including alkaloids, flavonoids, terpenes, phenolics and anthraquinones. The presence of synephrine, aspargine, glucose, fructose and fatty acids was revealed by GC–MS analysis. FB administration led to significant improved memory retention when evaluated through passive avoidance (*p* < 0.05), Y-maze (*p* < 0.05) and Morris water maze (*p* < 0.05) tests in a scopolamine model of amnesic rats. When tested by open field and elevated plus maze tests, FB demonstrated anxiety-resolving characteristics (*p* < 0.05) as animals dared to stay in open areas more than a control group. Mobility time was increased and immobility time was reduced (*p* < 0.05–0.01) in rats treated with FB, unveiling the anti-depressant importance of *F. benghalensis*. *Conclusion:* methanolic extract of *F. benghalensis* bark furnished scientific proof behind folkloric claims of the memory improving, anxiety-reducing and depression-resolving characteristics of the plant. These activities might be possible due to interaction of its phytoconstituents with serotonergic, glutamatergic, cholinergic and GABAergic systems in the brain.

## 1. Introduction

Every sixth individual of the world’s population suffers from some neurological disorder at least once in their lifetime. World Health organization (WHO) reports that approximately 450 million humans have mental disorders worldwide, thus placing them amongst the major detrimental ailments [1]. Alzheimer’s disease (AD), anxiety and depression are observed as the most prominent mental disorders that influence patients as well as their families [2]. Alzheimer’s disease comprises gradual development of neurodegeneration leading to physiological and cognitive decline [3]. Contributing pathophysiological factors are extracellular deposition of insoluble β-amyloid clumps, intracellular neurofibrillary tangles and oxidative stress followed by neuronal degeneration and impaired neurotransmission in the hippocampus [4]. Cholinergic neurotransmission is vital in memory formation and cholinergic hypothesis states that about 75% of cholinergic neurons are dramatically diminished by the last stages of AD [5]. Regaining cholinergic functionality by acetylcholinesterase inhibitors is mainstay as therapeutic option to cope with AD but short half-lives of these agents as well as their adverse effects provide the reason to look for alternative opportunities to treat this disorder [6].

Anxiety is one of most prevalent psychiatric disorder and currently 7.3% of the population is affected by it throughout the world [7]. This disorder comprises dreadful threats and complicated emotions of confusion and uneasiness [8]. Different neurotransmitters are present in the central nervous system (CNS) and regulation of neuronal activity results from a balance between excitatory (glutamatergic) and inhibitory (GABAergic) inputs. Even a mildest lessening of GABAergic activity can result into insomnia and anxiety. Benzodiazepines act by augmenting GABAergic inhibition and have prime importance as anxiolytic agents but possess misuse potential and life-threatening adverse effects [9]. Selective serotonin reuptake inhibitors (SSRIs) are becoming popular to treat anxiety but the doses required are larger than those required to treat depression. Additionally, their use can lead to suicidal risks, worsening behavior and withdrawal syndrome [10].

Depression is a mood disarray in which someone experiences symptoms like joylessness, insignificance, declined attentiveness, unhappiness, worthlessness, aggravation and irritability [11]. About 1 in 5 people are reported to experience one episode of depression and females are two times more prone to this than males [12]. The main underlying causes are brain circuits dysregulation [13], deficiencies of monoamine neurotransmitters [14], imprecise neuronal plasticity with decreased neurogenesis [15], atrophy of hippocampus, and reduced levels of glial fibrillary acidic-protein in the regions of the prefrontal cortex of the brain [16]. The discovery of antidepressant drugs brought a revolutionary reduction of suicidal risk in patients [17]. Limitations including low remission [18], poor tolerability [19] and pronounced side effects [20] are compelling evidence to look for some alternative therapy to rescue depressed patients. Recent studies report that 80% of the community in growing countries still trust on herbal remedies, as safe outcomes and cost efficacy have captured the minds of turning individuals back to nature [21].

*Ficus benghalensis* Linn. locally famous as Bargad in Pakistan, is one of the evergreen trees that has been widely utilized in eminent native remedies i.e., Unani, Ayurveda, Siddha and homeopathy [22]. Recent scientific studies have demonstrated anti-inflammatory potency on adjuvant induced arthritis [23] and antioxidant effects of *Ficus benghalensis* [23]. When tested in albino mice, extracts of aerial roots demonstrated better cognition, muscular relaxation and seizure reduction [24]. Different parts of *Ficus benghalensis* L have been known traditionally to resolve ailments related to the central nervous system [25] and many researchers have described various neuropharmacological effects of this plant [26]. These marvelous medicinal characteristics may be linked with phytoconstituents possessed by *Ficus benghalensis* including phytosterols [27], phenols [23], flavonoids [28] and terpenes [29].

The current study is planned to examine the presence of phytocomponents in *F. benghalensis* bark and outcomes of its methanolic extract of on memory, depression and anxiety in rats. We hoped to establish scientific proof to validate the usefulness of this plant in the aforementioned neurological disorders.

## 2. Materials and Methods

### 2.1. Animals

Animals utilized in testing were Sprague Dawley (SD) rats of both sexes weighing 100–150 grams. Rats were purchased from National Institute of Health, Islamabad, Pakistan, and subsequently accommodated in the vicinity of animal house of Faculty of Pharmacy, Bahauddin Zakariya University, Multan. Animals were caged individually in hygienically controlled atmosphere regulated at 25 °C with ad libitum access to water and standard food comprising 21% protein and 60% carbohydrate constituents. Before conducting the test, animals were habituated to handling procedures for three days before in order to avoid stress induced by handling during procedure. All animal studies were permitted by the Department of Pharmacology Ethical Committee Bahauddin Zakariya University (BZU), Multan and were employed with the ethical committee number (EC/03-PHLP/2017, approved 16.03.2017) in accordance with instructions of the Institute of Laboratory Animal Resources (ILAR), Commission on Life Sciences, National Research Council (NRC, 1996).

### 2.2. Preparation of Plant Extract

We collected 1 kg of bark from the plant *Ficus benghalensis* Linn. located in Garden Town Multan. The plant was authenticated by Dr. Zafarullah Zafar, taxonomist from the Department of Botany, BZU, Multan and voucher (R. R. Stewart 192) was retained in a herbarium. After removing the adulterants, bark was sliced into small pieces which were subsequently subjected to shade drying. Later, dried pieces were crushed coarsely and drenched with 80% v/v aqueous solution of methanol for one week while mixing intermittently. Soaked material was passed through filtration and process was performed twice. Collected filtrate was subjected to evaporation and concentrated in rotary evaporator to procure a thick brownish extract (9% yields). Obtained extract was stored at –20 °C and was dissolved in distilled water accordingly on test day for investigation.

### 2.3. Drugs and Chemicals

Diazepam, Fluoxetine, Scopolamine, Piracetam and Isopropyl alcohol (TCI Chemicals, Tokyo, Japan) used in this research were of analytical grade and highly pure. Methoxyamine hydrochloride, pyridine, bis-N,O-trimethylsilyl trifluoroacetamide (BSTFA) and chlorotrimethylsilane (TMCS) were purchased from Sigma-Aldrich, St. Louis, MO, USA. Hexane and methanol were purchased from BDH VWR International Ltd Poole, BH 15 1TD, England. Deionized water was obtained with Milli-RO and Milli-Q Plus instrumentation from Millipore, Billerica, MA, USA.

### 2.4. Identification of Phytocomponents

#### 2.4.1. Gas Chromatography–Mass Spectrometry (GC–MS) Analysis

Before the commencement of mass spectrometry, the crude sample was subjected to preliminary phytochemical analysis. A Perkin Elmer model Clarus 600 T (Perkin Elmer Waltham, Massachusetts, USA) combined with a single quadrupole mass spectrometer was used for gas chromatography–mass spectrometry (GC–MS) analysis. The chromatographic column was an Elite 5MS column by Perkin Elmer Waltham, Massachusetts, U.S.A (30 m × 0.25 mm × 0.25 µm film thickness), with high-purity helium as the gas carrier, at a flow rate of 1 mL/min. The injector temperature was 280 °C and it was equipped with a splitless injector at 20:1. The temperature was set initially to 40 °C (held for 2 min), then was increased to 150 °C at 10 °C per min (held for 2 min), then increased further to 300 °C at 10 °C per min for 2 min. The MS ion source temperature was 220 °C and inlet line temperature was set to 240 °C. The scan range was set at 40 to 600 mass ranges at 70 eV electron energy and the solvent delay of 4/min. Finally, unknown compounds were identified by comparing the spectra with that of the NIST 2005 (National Institute of Standard and Technology library) and the Wiley 2006 library. The total time required for analyzing a single sample was 32 min.

#### 2.4.2. Derivatization

The extracted plant samples were thawed at room temperature and vortex for 2 min. We filtered the extract at 0.45 micron and pipette out 300 μL then transferred it into a GC–MS vial. We purged the solution with nitrogen air to completely dry the vial. After this process methoxymation was carried out at room temperature by adding 100 μL of methoxyamine hydrochloride in pyridine solution (15 mg/mL). The mixture was vortexed for 10 min and put the sample for 16 h at room temperature. In the second step, the methoxymated sample underwent a derivatization reaction by using 100 µl of BSTFA/TMCS (99/1, v/v) and vortexed again for 10 min and kept for 2 h at 50 °C to complete the derivatizing reaction. 1 μL of the derivatized sample was injected into the system with the split mode (split ratio 1:20).

### 2.5. Behavioral Tests for Memory and Learning

#### 2.5.1. Passive Avoidance Task (Step-Through)

The equipment for this test comprises one dark compartment (20 × 20 × 20 cm) and one illuminated compartment (20 × 20 × 20 cm) connected through a door (5 × 5 cm) [30]. A bulb (100 W) was installed as source of light in illuminated section while floor comprising stainless steel rods were present in the dark section. Rats were placed in illuminated compartment during acquisition trials period for 10 s and the door was opened. Due to their innate behavior, rats instantly went into the dark compartment where they were provided with an electric foot shock (0.5 mA) for 3 s; 60 min before the acquisition trial, rats were administered with test compound *Ficus benghalensis* (FB) bark extract (100, 200, 300 mg/kg) or piracetam (200 mg/kg). Amnesia was induced by administering scopolamine 30 min after administration of the FB bark extract and piracetam in respective groups. Control group was treated with 10% Tween 80 solution only. Rats were again placed in lighted area after 24 h for retention trials. Step-through latency was measured as the delay to enter the dark compartment.

#### 2.5.2. Y-maze Task

This behavioral test is carried out in a Y shaped maze (50 × 10 × 15 cm) comprising three arms inclined at 120° to each other. Rats were administered with test compound FB bark extract (100, 200, 300 mg/kg) or piracetam (200 mg/kg) 60 min before the trial. Amnesia was induced by administering scopolamine 30 min after administration of FB bark extract and piracetam in respective groups. Control group was treated with normal saline (10 mL/kg) solution only. Rats were placed in one of three arms of maze to move freely and the sequence of arm visits (A, B and C) with the total number of each arm entries was monitored for 10 min [31]. Rats with intact memory preferred the new arm during their movement as they remembered the arms previously visited. This preference results in a number of triads which affects their alternation behavior. Greater percentage alternation behavior meant larger spatial working memory was gained by the rat; the percentage spontaneous alternation is calculated by using following formula:

% Spontaneous Alternation (%SAP) = [(Number of alternations)/(Total arm entries−2)] ×100.(1)

The number of arm entries serves as an indicator of locomotor activity.

#### 2.5.3. Morris Water Maze Task

The equipment of this behavioral study comprised a grayish spherical tank (150 cm diameter and 50 cm height) with impeccable internal surface [32]. The tank was filled up to 32 cm with water consisting opacifying white non-hazardous dye to disguise the platform. A square plastic platform (10 × 10cm) was submerged under 1–2 cm of water in the middle of one of the quadrants [33]. Distal hints comprising a few geometrical signs were displayed around the pool [34] and proximal signs were illustrated inside the tank keeping the arrangements constant throughout the studies. On the first day, rats were prepared to swim in the pool without the platform for 60 s. For the next three days, rats were tested in the pool with the platform where they were subjected to two trials in every 24 h keeping minimum inter-trial gap of 30 min. If a rat successfully reached the platform, he was allowed to stay there for 30 s or he was slightly pushed towards the platform in the case of failure to locate it within 120 s. The latency to locate the platform was recorded using a Logitech HD camera. One day subsequent to last training session, platform was taken out permitting rats to search it for 120 s. Spatial learning is acquired if they are frequently visiting the quadrant where platform was previously situated [35]. piracetam (200 mg/kg, i.p.) was administered as positive control 1 h before the first trial on each day. Cognitive impairment was induced by scopolamine (1 mg/kg, i.p.) at 30 min after treatment with various doses of extract (100, 200 and 300 mg/kg) and piracetam.

### 2.6. Behavioral Tests for Anxiety

#### 2.6.1. Open Field Test

A novel scientific procedure was employed to assess the exploration behavior in rodents and to correlate them with anxiety-resolving potential of plant extracts [36]. Equipment was a square-shaped field (80 × 80cm) composed of polyacryline with 40 cm high walls to prevent escape. The experimental room was satisfactorily quiet and lit. About 60 min before the experiment, rats of designated groups were given the test compound (FB 100, 200 and 300 mg/kg, intraperitoneal (i.p.)) and negative control (normal saline 10 mL/kg, i.p.). Diazepam (2 mg/kg, i.p.) was given in positive control group 30 min before test. Animals were monitored for 5 min and the number of corner and center zone entries with respective duration and number of line crossings were noted.

#### 2.6.2. Elevated Plus-Maze Test

This behavioral study was executed to evaluate the affectivity of test compounds to resolve anxiety [37]. This test was based upon the instinct behavior of rodents to avoid risky surroundings (open arm) and to prefer a safe environment (close arm) [38]. The “+” shaped apparatus comprised four arms (110 × 10 cm), two open and two closed, mounted 50 cm above from base. Animals were administered with a drug and were subjected to testing 30 min later. Rats were positioned in the center of the maze in such a way that they were facing open arms. Parameters such as open and close arm durations and number of open arm entries were observed. Increased entries in open arms and increased time spent there were taken as anxiety-free behavior in rats.

### 2.7. Behavioral Tests for Depression

#### Forced Swimming Test

This is a very reliable and widely used behavioral test for the prediction of effective antidepressant potential of treatments [39]. Apparatus was a deep tank (34.5 × 23.5cm) made up of glass and comprising water (25 °C) to the mark so that the rat could not touch the tank bottom. For this test, animals were divided in 5 groups each comprising 6 rats. Three experimental groups received 100, 200 and 300 mg/kg of extract, respectively, an hour before initiating test and results were compared with fluoxetine (20 mg/kg i.p.) and control (normal saline group 10 mL/kg, i.p.) groups. Each rat was gently released in the center of the water tank and was enforced to swim. The duration of mobility and immobility phases was recorded. Enhanced swimming behavior and reduced immobility were signs of the antidepressant potential of the test compound.

### 2.8. Statistical Analysis

One-way analysis of variance (ANOVA) followed by Tukey’s multiple comparison tests was used for statistical analysis of almost all parameters of behavioral activities. However, Two-way ANOVA followed by Tukey’s test was employed for step through latencies, time spent in dark and escape latencies in the passive avoidance test and Morris water maze test respectively.

## 3. Results

### 3.1. Phytochemical Analysis

Preliminary phytochemical studies confirmed the presence of alkaloids, proteins, flavonoids, saponins, carbohydrates, steroids, phenols, tannins, carbohydrates and terpenes.

### 3.2. GC–MS Analysis for Phytocomponents

**Figure 1:**
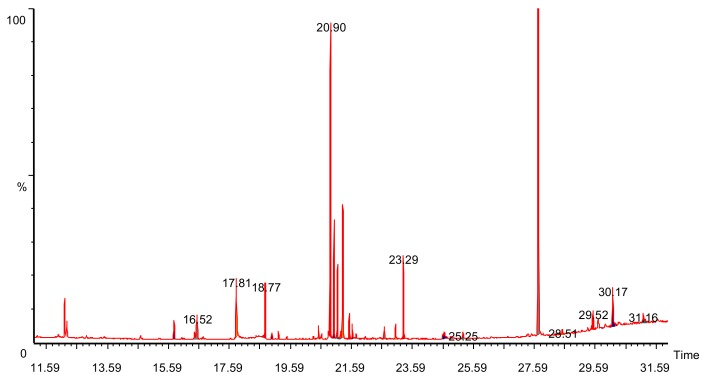
Total ion chromatogram (TIC) of *Ficus benghalensis* bark.

GC–MS analysis showed the presence of alkaloids like synephrine, amino acids like aspargine, higher fatty acids and carbohydrates like glucose and fructose (Figure 1). The retention time of the respective compound and relative percentage of area is reported in Table 1.

### 3.3. Effect on Spatial Memory and Learning

#### 3.3.1. Passive Avoidance Test

**Figure 2:**
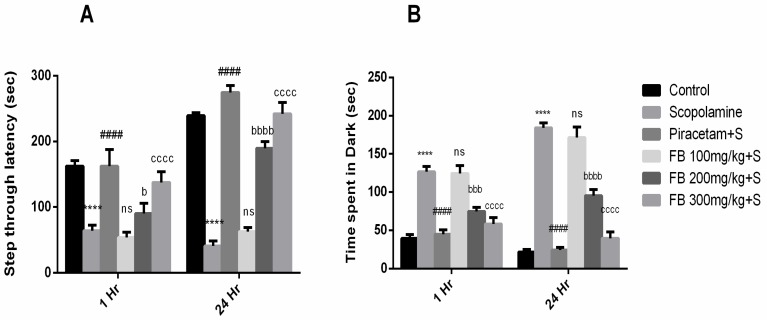
Impact of *Ficus benghalensis* (FB) bark extract (100, 200 and 300 mg/kg) on scopolamine (2 mg/kg, intraperitoneal (i.p.)) treated rats on step through latencies (**A**) and time spent in dark (**B**) during passive avoidance test using Piracetam as positive control. Data are presented as ± standard error of the mean (SEM) (*n* = 6 per group). **** *p* < 0.0001 comparison between control (vehicle treated) and Scopolamine group, ^####^ *p* < 0.0001 comparison between scopolamine and piracetam treated groups. ^b^ *p* < 0.01, ^bbb^ *p* < 0.001, ^bbbb^ *p* < 0.0001 comparison between scopolamine and FB 200 mg/kg treated groups. ^cccc^ *p* < 0.0001 comparison between scopolamine and FB 300 mg/kg treated groups. ^ns^ not significant.

Our results demonstrated that *F. benghalensis* bark extract improved learning and memory retention in rodent amnesic model induced by scopolamine. Step-through latency to enter the dark section of equipment after 1 and 24 h by scopolamine-treated rats was notably shorter than the vehicle treated group (*p* < 0.001). In the piracetam (positive control) treated group, latency was much higher compared to the scopolamine group (Figure 2A). Additionally, shorter latencies by scopolamine were reversed by pretreatment with 200 mg/kg and 300 mg/kg of FB bark (*p* < 0.05–0.0001).

Moreover, time spent in darkroom by amnesic rats was considerably higher than the vehicle treated group (*p* < 0.0001). Treatment by 200 and 300 mg/kg of FB bark extract lessened this time compared to the scopolamine group (*p* < 0.001–0.0001). However, at a dose of 100 mg/kg, FB bark extract did not exhibit a noteworthy effect (Figure 2B).

#### 3.3.2. Y-maze Test

**Figure 3:**
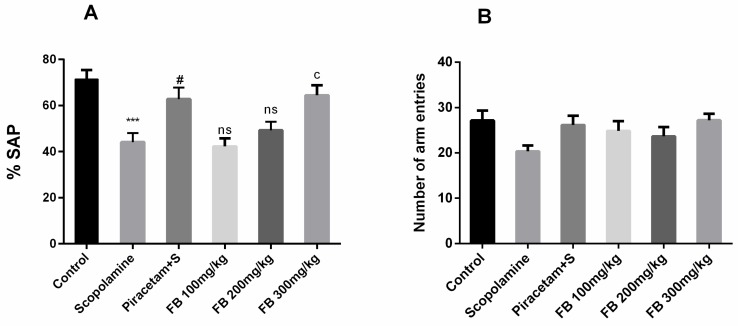
Impact of FB bark extract (100, 200 and 300 mg/kg) on Scopolamine (2 mg/kg, i.p.) treated rats on % spontaneous alternation (**A**) and number of arm entries (**B**) during Y-maze task using piracetam as positive control. Data are presented as ± SEM (*n* = 6 per group). *** *p* < 0.001 comparison between control (vehicle treated) and scopolamine group, ^#^ *p* < 0.05 comparison between scopolamine and piracetam treated groups.^c^ *p* < 0.05 comparison between scopolamine and FB 300mg/kg treated groups. ^ns^ not significant.

The effect of FB bark extract on spontaneous alternation behavior was inspected using the Y-maze test. Spontaneous alternation behavior was significantly lower in the scopolamine group compared to vehicle-treated group (*p* < 0.001) and these effects were reversed by FB 300 mg/kg (*p* < 0.05) and as a result %SAP (spontaneous alternation) score was comparable to that of piracetam (Figure 3A). However, outcomes by remained insignificant at doses 100 and 200 mg/kg. Anyhow, total arm entries were quite similar among all groups indicating that the locomotor activity of rats was not affected (Figure 3B).

#### 3.3.3. Morris Water Maze Test

**Figure 4:**
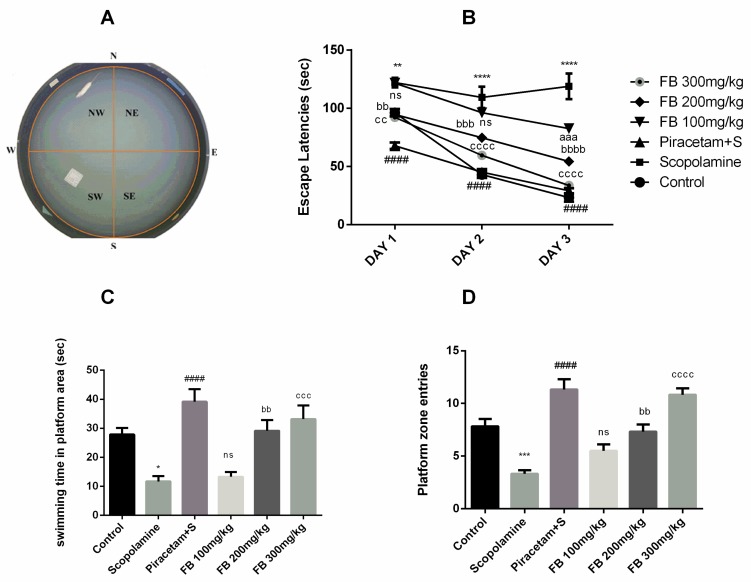
Display of proximal cues in Morris water maze (MWM) tank (**A**), impact of FB bark extract (100, 200 and 300 mg/kg) on scopolamine (2 mg/kg, i.p.) treated rats on escape latencies from day 1 to 3 (**B**), swimming time in platform area (**C**) and platform zone entries (**D**) on probe day using piracetam as positive control. Data are presented as ± SEM (*n* = 6 per group). * *p* < 0.05, ** *p* < 0.01, *** *p* < 0.001, **** *p* < 0.0001 comparison between control (vehicle-treated) and scopolamine group, ^####^ *p* < 0.0001 comparison between scopolamine and piracetam treated groups. ^aaa^ *p* < 0.001 comparison between scopolamine and FB 100 mg/kg treated groups. ^bb^ *p* < 0.01, ^bbb^ *p* < 0.001, ^bbbb^ *p* < 0.0001 comparison between scopolamine and FB 200 mg/kg treated groups. ^cc^ *p* <0.01, ^ccc^ *p* < 0.001, ^cccc^ *p* < 0.0001 comparison between scopolamine and FB 300 mg/kg treated groups. ^ns^ not significant.

The Morris water maze was used to evaluate the effects of *F. benghalensis* bark extract on spatial learning. During three consecutive training days, vehicle-treated rats were able to find the hidden platform more quickly than those treated with scopolamine. Scopolamine administration produced longer escape latencies compared to the vehicle-treated group from days 1 to 3 (*p* < 0.01–0.0001). Piracetam shortened the escape latencies which were prolonged by scopolamine (*p* < 0.0001). *Ficus benghalensis* bark extract also decreased the escape latencies at doses of 100, 200 and 300 mg/kg compared to the scopolamine-treated group (*p* < 0.01–0.0001) (Figure 4B).

The probe day results showed that number of entries and swimming time in the quadrant where the platform was previously present were significantly decreased by scopolamine compared to the vehicle-treated group (*p* < 0.001) and (*p* < 0.05) respectively. But these outcomes were reversed by *F. benghalensis* bark extract (Figure 4C and 4D) at 200 and 300 mg/kg (*p* < 0.01–0.0001) in a similar way to piracetam when compared to scopolamine group (*p* < 0.0001).

### 3.4. Anxiolytic Effect

#### 3.4.1. Open Field Test

**Figure 5:**
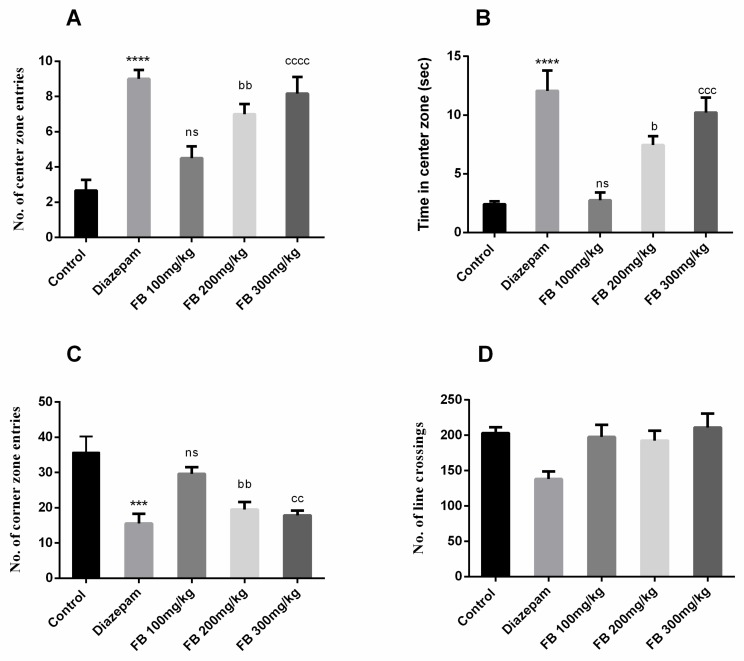
Effect of FB bark extract (100, 200 and 300mg/kg) as anxiolytic on number of central zone entries (**A**) and time in center zone (**B**), number of corner zone entries (**C**) and number of line crossings (**D**) in open field test using diazepam as positive control. Data are presented as ± SEM (*n* = 6 per group). *** *p* < 0.001, **** *p* < 0.0001 comparison between control (vehicle-treated) and diazepam group, ^b^ *p* < 0.05, ^bb^ *p* < 0.01, comparison between control and FB 200 mg/kg treated groups. ^cc^ *p* < 0.01, ^ccc^ *p* < 0.001, ^cccc^ *p* < 0.0001 comparison between control and FB 300 mg/kg treated groups. ^ns^ not significant.

The open field test was used to evaluate anti-anxiety effects of FB bark extract. Animals prefer corners when exposed to open areas as evident from the behavior of rats in the control group. However, significant difference in number of center zone entries (F (4, 25) = 14.91, *p* < 0.0001), time in center zone (F (4, 25) = 16.38, *p* < 0.0001) and number of corner zone entries (F (4, 25) = 9.767, *p* < 0.0001) between all groups was described by ANOVA as depicted in Figure 5 (A), (B) and (C). Furthermore, Tukey’s post hoc test revealed that the numbers of entries in center and corner zones were significantly different between control and diazepam (2 mg/kg) groups (*p* < 0.001–0.0001). The groups treated with 200 and 300 mg/kg of FB bark extract showed increased center zone entries (*p* < 0.01–0.0001) and decreased corner zone entries (*p* < 0.01) compared to control groups. Similarly, prolonged time was spent in the center zone by FB (200 and 300 mg/kg) treated rats (*p* < 0.05–0.001). However, the number of crossings were decreased by diazepam only and remained unaffected in FB treated groups (Figure 5D). No beneficial outcomes were obtained by FB bark extract at a dose of 100 mg/kg.

#### 3.4.2. Elevated Plus Maze Test

**Figure 6:**
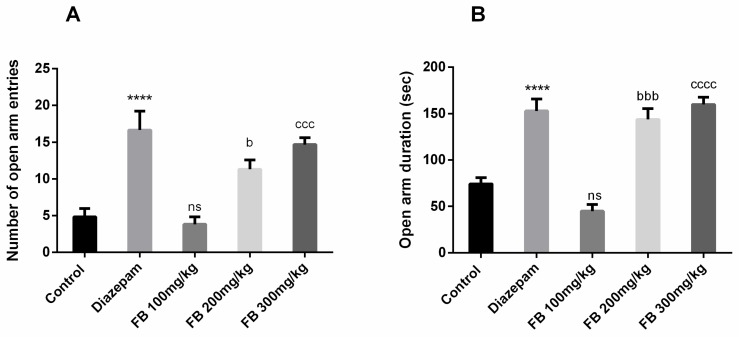
Impact of FB bark extract (100, 200 and 300 mg/kg) as anxiolytic on open arm entries (**A**), and open arm duration (**B**) in elevated plus maze test using diazepam as positive control. Data are presented as ± SEM (*n* = 6 per group). **** *p* < 0.0001 comparison between control (vehicle-treated) and diazepam group, ^b^ *p* < 0.05, ^bbb^ *p* < 0.001 comparison between control and FB 200 mg/kg treated groups. ^ccc^ *p* < 0.001, ^cccc^ *p* < 0.0001 comparison between Control and FB 300 mg/kg treated groups. ^ns^ not significant.

FB bark extract was tested for anxiolytic effects using the elevated plus maze test. Results revealed significant difference of number of open arm entries (F (4, 25) = 14.61, *p* < 0.0001) and open arm duration (F (4, 25) = 29.76, *p* < 0.0001) among all groups was revealed by ANOVA (Figure 6 A and B) Innate behavior of rats to show anxiety when exposed to an elevated maze was seen in the control group but the administration of FB bark extract reversed the anxiety in a dose-dependent manner and outcomes were not significant at doses of 100 mg/kg.

### 3.5. Antidepressant Effect

#### Forced swimming test

**Figure 7:**
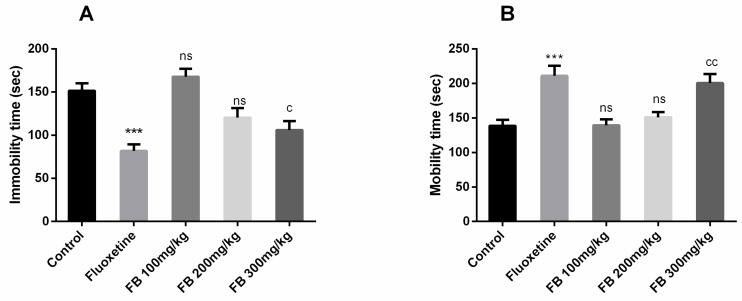
Effect of FB bark extract (100, 200 and 300 mg/kg) as antidepressant on immobility time (**A**) and mobility time (**B**) in forced swimming test using Fluoxetine as positive control. Data are presented as ± SEM (*n* = 6 per group). *** *p* < 0.001 comparison between control (vehicle-treated) and fluoxetine group, ^c^ *p* < 0.05, ^cc^ *p* < 0.01 comparison between control and FB 300 mg/kg treated groups. ^ns^ not significant.

In this test, significant differences of immobility time (F (4, 25) = 13.20, *p* < 0.0001) and mobility time (F (4, 25) = 10.33, *p* < 0.0001) among all groups were revealed by ANOVA as shown in Figure 7(A) and (B). Moreover, Tukey’s post hoc test detected significant differences between control vs. fluoxetine (20 mg/kg) treated group (*p* < 0.001) and control vs. FB 300 mg/kg treated groups (*p* < 0.05) for the mobility time. Tukey’s post hoc test revealed a significant difference between control vs. fluoxetine treated group (*p* < 0.001) and control vs. FB 300 mg/kg treated groups (*p* < 0.05) for the mobility time. Immobility and mobility remained unaffected on FB bark doses of 100 and 200 mg/kg but at a dose of 300 mg/kg, FB bark reduced immobility and increased mobility as did the fluoxetine.

## 4. Discussion

In developing countries, a significant proportion of people rely on natural remedies for amelioration of various neurological disorders based on their affordability and the limited availability of advanced medical infrastructure. To overcome such barriers towards a healthy life, researchers have been continuously attempting to discover therapeutic potentials of remedial plants used historically [40]. A range of phytoconstituents present in plants, i.e., alkaloids, amino acids and carbohydrates, might be responsible for these medicinal characteristics [41]. In the current study, the neuroprotective potential of *F. benghalensis* bark was assessed by conducting behavioral studies in rats. We employed the Morris water maze test, Y-maze test and passive avoidance test to evaluate learning and memory which progressively diminish by the normal aging process as well as due to certain disorders i.e., Alzheimer’s disease. Open field and elevated plus maze assessment were utilized to evaluate anxiety while the forced swimming experiment was employed to evaluate depression-like behavior in rats.

Cholinergic neurons in the brain are fundamentally significant in memory formation and their disruption leads to Alzheimer’s disease [42]. Scopolamine, a muscarinic antagonist, impairs reference (long-term), working (short-term) memories as well as learning capabilities in humans and animals [43]. In the present study, scopolamine was used to disrupt memory in rats and impact of *F. benghalensis* bark on this deteriorated memory and learning was evaluated. The conclusion of all tests utilized provided the evidence that FB bark extract reverses memory deteriorated by scopolamine. 

The passive avoidance test is based on a concept that rodents tend to prefer dark places but the application of unavoidable electric shock leads to suppression of this innate behavior as the animal retains memory of a noxious event and attempts to avoid it [44]. Scopolamine administration led to decreased latency to go into dark section and increased time spent there even after the shock revealing memory-deteriorating characteristics of scopolamine [45]. These parameters were significantly reversed by FB bark extract in a dose-dependent manner. Y-maze did further confirm these outcomes on hippocampus-dependent spatial memory [10]. This test is formulated on some inbuilt characteristics of rodents to discover novel surroundings [46]. In this experimental model, percentage spontaneous alternation, parameter to reflect short-term spatial memory is recorded [47]. Acute administration of 300 mg/kg of FB led to significantly increased %SAP in comparison to scopolamine group. It is also valuable to point out that overall arm entries amongst every group remained unchanged revealing that any locomotor impairment had no influence on the observed results. The Morris water maze task, hippocampus-dependent test, was also employed in study to validate the spatial memory enhancing capability of test compound. Scopolamine administration induced memory impairment as evidenced by prolonged escape latencies in the scopolamine-alone treated group. FB extract significantly reversed these effects at all administered doses across three days. On probe day, duration and entries in the platform region were also increased by 200 and 300 mg/kg FB doses. If rats roamed for lengthy distances and spent more time in the platform zone, they acquired spatial memory improvement in the Morris water maze (MWM) task [48].

These remembrance-improving characteristics of FB bark might be due to the presence of flavonoids as they promote blood flow to brain that contributes towards enhanced memory and learning [49]. Another feature of flavonoids is they may inhibit the acetylcholinesterase enzyme [50] thus there is increased acetylcholine in the hippocampus [51]. Acetylcholine, in turn, dampens the immune response of the brain by down streaming the actions of both neurons as well as receptors glial cells [52] which if activated are a hallmark of brain pathology [53]. Flavonoids may also improve memory by binding and activating CREB proteins (cAMP response element-binding proteins) that promote the expression of genes linked with memory and strengthen neurotransmission and flow of information [54]. CREB also affects functioning by affecting the assembly of neutrophins-protein [55]. Another important constituent of FB bark are phytosterols as they play imperative role in modulating amyloid precursor protein (APP) processing and amyloid beta plaque formation which are molecular activities contributing towards dementia [56]. Although other neuroprotective inputs by the phytosterols such as anti-oxidant, and cholinergic modulation inputs cannot be ignored.

In the current study, we also observed prominent anxiolytic effects in rats after acute administration of FB bark extract at 200 and 300 mg/kg doses. Open field test and elevated plus maze tests have been taken up by many researchers to investigate exploratory and as anxiety index in rodents. Anxiety was reduced by *Ficus benghalensis* bark extract as evidenced by increased entries and time spent in the central zone while corner entries were also reduced. These anxiety-resolving characteristics of extract were further authenticated by elevated plus maze test. Outcomes revealed that visits of open arms as well as duration spent there were increased in rats at higher doses. Diazepam, a GABA agonist, was used as positive standard in both tests which augments neuronal inhibition by elevating GABA in the brain thus exerting anxiolytic effects. It is worthy to mention that FB bark extract did not affect the number of crossings in open field test (OFT) explaining that it had no effect on locomotor activity of rats as observed in the Y- maze also. Thus, it can be claimed that FB bark has the potential to resolve anxiety without having sedative effects. This anti-anxiety potential of FB bark might be due to the synergistic effect of certain phytoconstituents present in extract [57]. Many lines of scientific studies have reported on the flavonoid’s high affinity for GABA receptors and therefore strongly modulates GABAergic system in brain [58]. 

The forced swimming test is accepted as reliable behavioral test to predict locomotor and antidepressant potential of treatment [59]. In this test, bark extract was assessed for antidepressant activity using different parameters. Fluoxetine was used as a positive standard which is an inhibitor of serotonin transporter protein, thus serotonin uptake from the synaptic cleft back to the presynaptic neuron is inhibited [60]. Results demonstrated that rats treated with 200 and 300 mg/kg doses demonstrated decreased immobility time and increased swimming time revealing that extracts possess antidepressant activity. This depression resolving potential of extract is possibly due to multiple phytoconstituents which might be acting through various mechanisms to enhance central noradrenergic and/or serotonergic neurotransmissions. Brain-derived neutrophic factor (BDNF) is a neurotrophin which is important for neuronal protection and survival [61] and has been involved in neurological disorders. A decreased level of BDNF is among most validated biomarkers of depressive disorder. BDNF levels are increased in the hippocampus of treated animals due to flavanones, thus unveiling their role as antidepressants [62]. Flavonoids have also been known to inhibit monoamine oxidases which are involved in the breakdown in monoamines and resulted in alterations in monoaminergic transmission has been seen in psychiatric disorders such as depression and anxiety. Moreover, monoamine oxidases also take part in the generation of hydrogen peroxide that leads to oxidative cell damage. These phenolic and flavonoid compounds in FB bark play a neuroprotective role as they exhibit pronounced antioxidant activity. Oxidative stress has been associated with the pathogenesis of several diseases including various psychiatric disorders [63]. Depression and anxiety are seen to be related to lowered levels of few amino acids and vitamins having antioxidant potential [64]. There is abundant evidence that plants rich in flavonoids are highly efficient in blocking such oxidation-induced neuronal damage [65]. Furthermore, flavonoids instruct anti-apoptotic actions by preventing the activation of caspase-3 and block oxidative-induced neuronal damage [66].

Aging, stress, anxiety and depression have been suggested to hinder adult hippocampal neurogenesis [67]. Polyphenols and flavonoids promote neuronal proliferation thus are important for adult hippocampal neurogenesis [68]. They might do so by activating or stimulating the expression and release of neurotrophic factors [68]. Phytosterols are capable of modulating various endothelium-dependent biological activities responsible for CNS disorders i.e., ischemia–reperfusion, vasorelaxation, neuroinflammation and oxidative stress [69].

Chemical profiling of crude extract (FB) revealed the presence of synephrine, an alkaloid that exerts an inhibitory effect on acetylcholinesterase and butyrylcholinesterase enzyme (BuChE) [70]. Hence, hydrolysis of acetylcholine is lessened and neuronal cholinergic transmission is improved by synephrine thus leading to improved cognition and learning [71]. Beside these, increased BuChE is also claimed to promote conversion of “benign” plaques to “malignant” plaques eventually leading to neuronal degeneration [72] and AD progression [73]. Additionally, synephrine might act as an α1-receptor agonist [74] which are widely spread in various regions of brain and may modulate different behavioral activities [75]. Various researchers have stated that synephrine decreased immobility time [74] and increased swimming time in rodents [74,76]. These anti-depressant outcomes may be possible due to α1-receptor activation by synephrine [77]. Hence, its presence in bark extract could be responsible for the aforementioned neuromodulatory outcomes of the current study.

## 5. Conclusion

The present study revealed neuromodulation effects of FB bark extract. Acutely administered doses of extract improved learning and memory as well as producing anxiolytic and antidepressant effects with no sedative side effect in rats. These outcomes provide the pharmacological evidence of folkloric uses of this plant for some neurological disorders. These potentials are possibly due to presence of beneficial phytochemicals in *Ficus benghalensis*. However, the exact mechanism behind these actions needs to be investigated in future.

## Figures and Tables

**Table 1:** Phytocomponents identified in methanol extract of *Ficus benghalensis* bark.

S.No	Phytocomponents	RT	Area%	Area
1	Butedioic acid	15.77	2.230	507,182
2	Ribitol	16.3	0.350	79,119
3	Butanoic acid	16.52	1.710	387,201
4	L-asparagine	18.77	3.150	714,501
5	Synephrine	18.97	0.590	133,192
6	Adonitol	19.19	0.610	138,566
7	Azelaic acid	20.34	0.220	50,773
8	Citric acid	20.51	0.960	218,233
9	Cyanuric acid	20.90	24.53	5,570,956
10	D-Fructose	21.02	7.33	1,665,137
11	Galactose	21.24	0.520	117,565
12	Glucitol	21.31	9.21	2,090,264
13	Glucose	21.52	1.65	37,4594
14	Adonitol	21.75	0.49	11,0464
15	Inositol	22.67	0.850	19,2694
16	Hexadecanoic acid	23.03	0.610	13,7463
17	Myo-inositol	23.29	5.13	117,5429
18	Trans-9-octadecanoic acid	24.64	0.38	86877
19	2-O-Glycerol alpha -D-Glucopyranoside	25.25	0.510	116,099
20	Alpha-D glucopyranoside	27.72	31.75	7,210,211
21	Turanose	28.51	0.25	56,761
22	Benzoic acid	29.52	2.59	588,105
23	Melibiose	30.17	3.46	784,751

RT—Retention time.
